# Metal Organic Spin Transistor

**DOI:** 10.1021/acs.nanolett.1c01865

**Published:** 2021-10-18

**Authors:** Naama Goren, Tapan Kumar Das, Noam Brown, Sharon Gilead, Shira Yochelis, Ehud Gazit, Ron Naaman, Yossi Paltiel

**Affiliations:** † Applied Physics Department and the Center for Nano-Science and Nano-Technology, The Hebrew University of Jerusalem, Jerusalem 91904, Israel; ‡ Department of Chemical and Biological Physics, Weizmann Institute, Rehovot 76100, Israel; § Department of Molecular Microbiology and Biotechnology, The Shmunis School of Biomedicine and Cancer Research, George S. Wise Faculty of Life Sciences, Tel Aviv University, Tel Aviv 6997801, Israel

**Keywords:** Organic memory, organo-metallic device, chiral-induced spin selectivity, spin transistor, multistate memory, spintronics

## Abstract

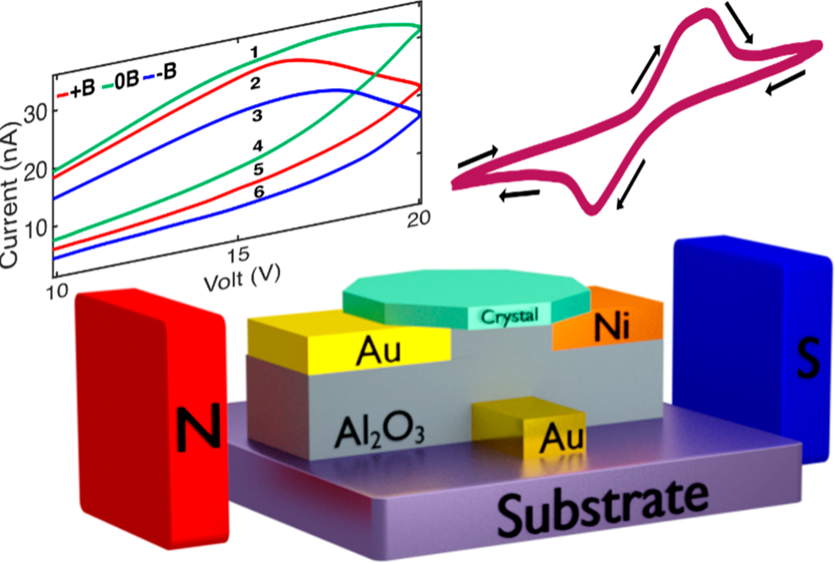

Organic molecules and specifically bio-organic systems are attractive for applications due to their low cost, variability, environmental friendliness, and facile manufacturing in a bottom-up fashion. However, due to their relatively low conductivity, their actual application is very limited. Chiral metallo-bio-organic crystals, on the other hand, have improved conduction and in addition interesting magnetic properties. We developed a spin transistor using these crystals and based on the chiral-induced spin selectivity effect. This device features a memristor type behavior, which depend on trapping both charges and spins. The spin properties are monitored by Hall signal and by an external magnetic field. The spin transistor exhibits nonlinear drain-source currents, with multilevel controlled states generated by the magnetization of the source. Varying the source magnetization enables a six-level readout for the two-terminal device. The simplicity of the device paves the way for its technological application in organic electronics and bioelectronics.

Organic materials are attractive for many applications due to their relatively low cost, the ability to control their properties,^1^ and their ease of manufacturing.^2,3^ Their development is also consistent with the tendency to avoid the use of toxic inorganic materials and other hazardous components. In addition, many organic systems possess the ability to be assembled in a bottom-up fashion using simple self-associating modules. Biological building blocks are especially attractive due to their inherent biocompatibility and safety as well as the ability to produce them in large numbers using natural systems. Furthermore, many biological modules can be readily assembled into well-organized structures with nanoscale order. Amino acids are one of the most attractive components of the biological world because of the ability to use them to form layered materials with unique chemical and physical properties.^4,5^

When organic electronics is considered, its main application is for organic light-emitting diodes (OLEDs).^6−8^ For memory applications, organic materials are very promising; however, they also suffer from having relatively low conductivity and large variability.^9,10^ In spintronics, organic materials have the advantage of having a relatively long spin lifetime; however, the low conduction and the need to interface organic materials with inorganic ferromagnetic electrodes make their use a challenge.^11,12^ Metal organics combine metal ions or clusters with organic ligands and can add magnetic and/or conductive properties. Here we present chiral metal–organic crystals (MOCs) as amino-acid-based conductive materials that also have attractive magnetic properties.^13,14^ We demonstrate the use of these crystals, which are chemically and structurally stable, as spin transistor devices.

In the last decades, spin-based devices have been utilized to achieve faster and higher density memories, with low power consumption.^15−19^ The ongoing challenge is to develop a simple technology that is dense, reliable, and fast.^20^ The chiral-induced spin selectivity (CISS) effect^21^ offers a unique approach to fabricate simple and small spintronics devices.^22^ Because of the CISS effect, chiral molecules and crystals can act as very efficient spin filters at room temperature.^23−28^ Recently, three-dimensional metal–organic frameworks were shown to behave as very efficient spin filters, with spin polarization reaching 100%.^27^ The CISS effect has been used to fabricate a spin-based magnetic memory device that operates without the need for a permanently magnetized ferromagnet.^29,30^ Similar experiments showed that photoexcitation of quantum dots, attached to a Hall device through a chiral self-assembled monolayer (SAM), can act as an optical memory device.^31−33^ By using chiral metal–organic bioinspired crystals, we utilized the CISS effect in fabricating MOCs.

The MOC we used consists of chiral Cu-phenylalanine crystals. In a recent work, these crystals were shown to have good conduction and both ferromagnetic and ferroelectric properties at room temperature.^13^ As a demonstration of their material properties, we showed that this material can be used to fabricate spintronic devices with multilevel memory^34^ as well as a spin transistor device.^35^ This spin transistor device also exhibits nonlinear memristive behavior utilized as a universal memory, and it enables logic operations.^36,37^

d-Enantiomers of the amino acid phenylalanine were crystallized with copper ions and characterized by X-ray analysis and circular dichroism (CD) spectroscopy. The asymmetric units of the crystal consist of a phenylalanine dimer coordinating a copper atom; the unit cell consists of two dimers, as seen in Figure 1a. Figure 1b and c presents high-order assemblies of the crystal viewed along different axes. Figure 1d and e presents scanning electron microscopy (SEM) and optical microscopy images, respectively.

**Figure 1:**
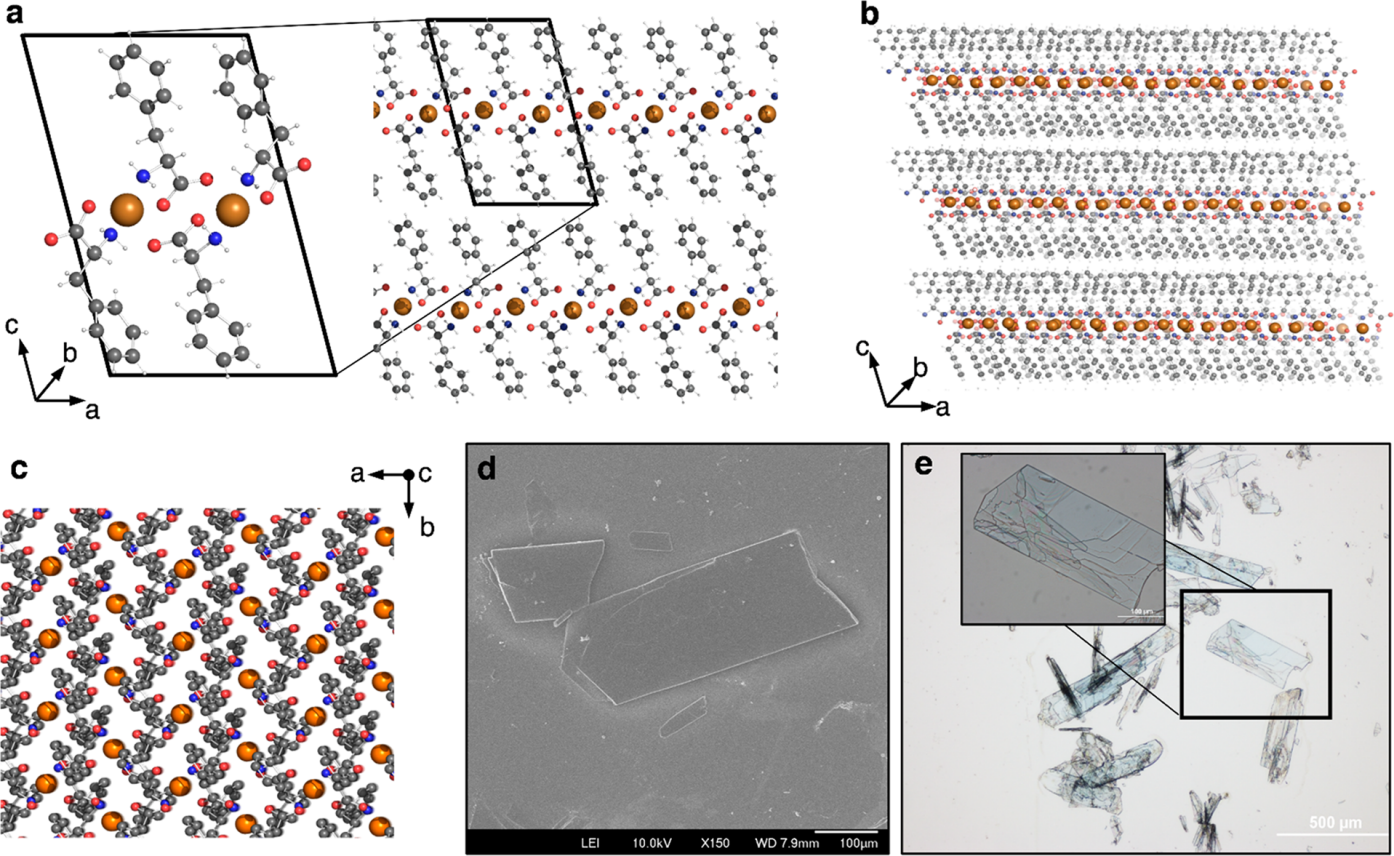
Structure of the d-phenylalanine-copper crystal. (a) Unit cell and a small view down the *b* axis. (b) High-order assembly demonstrating the layered structure of the crystal. (c) High-order assembly viewed along the *c* axis. (d) SEM micrograph. (e) Optical microscope image.

Figure 2a presents the absorption spectrum of the crystal. The crystal has strong optical activity in the UV region and low but not negligible absorption in the visible region. Figure 2b presents the CD spectrum of the crystal, whereas Figure 2c presents the absorption spectra when the crystal is excited with clockwise (RCL) and counterclockwise (LCL) circularly polarized light.

**Figure 2:**
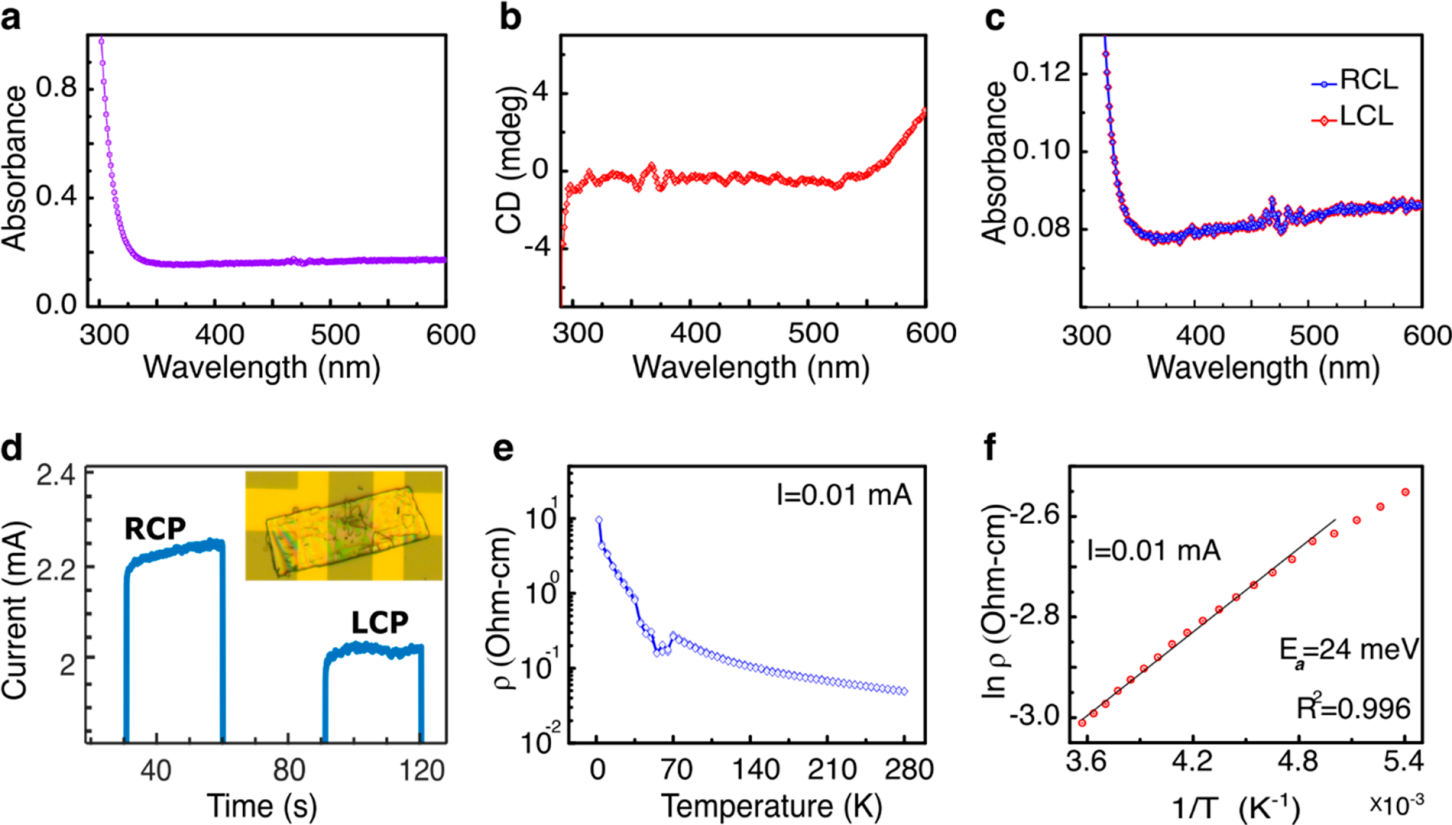
Crystal optical and electrical properties. (a) Absorption spectrum using linear polarized light. (b) CD spectrum of the crystal. (c) Absorption spectra under illumination measured with clockwise (blue-RCL) and counterclockwise (red-LCL) circularly polarized light. The electronic structure is presented in (d)–(f). (d) Photocurrent measured at a constant voltage of 5 V for right-handed circularly polarized (RCP) and left-handed circularly polarized (LCP) light. The full *I*–*V* curve is presented in Figure S6. Inset: Optical microscope image of the device with a right-handed chiral MOC placed between two Au pads (see also Figure S6). (e) Dependence of the resistivity on temperature in log scale. (f) Arrhenius plot presenting the resistivity versus the inverse temperature. The activation energy, *E*_a_, is obtained by multiplying the slope by the Boltzmann factor. It corresponds to the band gap in the crystals.

All electrical measurements were performed in a planar architecture when the device is located on a thermal oxide (SiO_2_-100 nm) p-type silicon wafer. An optical microscope image and a graphic sketch of the right-handed d-enantiomers of phenylalanine MOC crystals located between two gold pads are shown in the inset of Figure 2d, which also presents the photocurrent through the crystals measured at a bias of 5 V, for left or right circularly polarized light (532 nm). Clearly, the photocurrent is about 10% stronger for the right circular polarization, despite that the light absorption at this wavelength is almost identical for the two circular polarizations (Figures 2c and S6). It is important to note that chirality is the same, independent of the angle of observation. Therefore, the large dependence of the photocurrent on the light circular polarization must result from the preferred spin conductivity. It was previously found that conduction through the crystals is spin selective at room temperatures, due to the CISS effect.^13^ In that reference, the coercive field is measured at different temperatures and was found to be about 200 Oe at room temperatures. Since the excitation by circularly polarized light results in exciting in a one spin direction, following the excitation, there is an electron in the excited state with one specific spin and the hole in the ground state has the same specific spin. Using the two-band conduction model,^38^ we expect that the hole conduction will be improved as a result of the reduced scattering for the same spin. If this spin will be the spin preferred for conduction through the chiral system, then a large photocurrent will be observed.

A similar improvement in conductivity, after magnetization of the Cu atoms, is presented in Figure 3. For the opposite circular polarization excitation, the chiral-preferred conducted spin will face a spin blockade on the excited Cu ions and its conduction will be suppressed.

**Figure 3:**
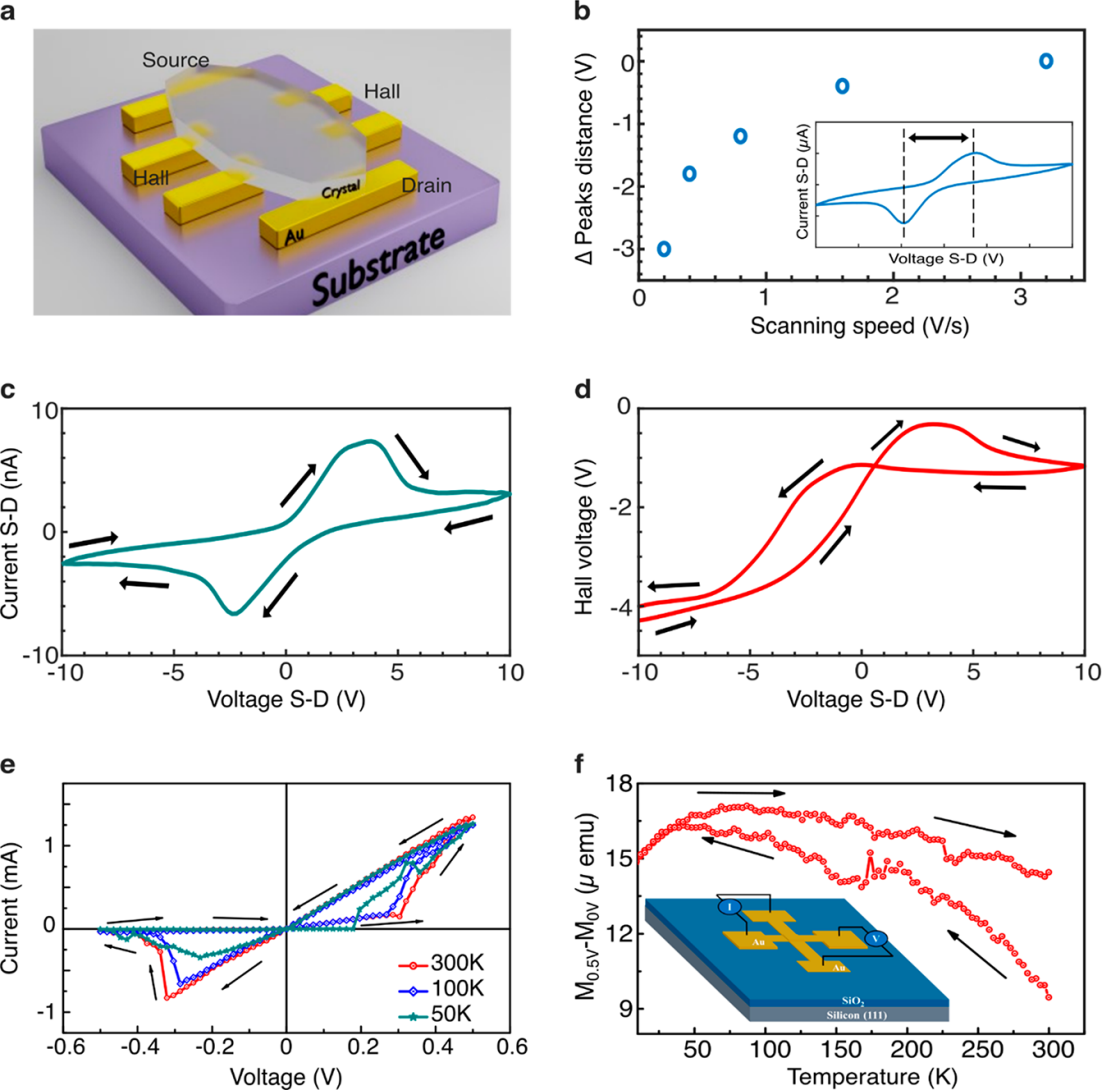
Spin transistor characteristics. (a) Sketch of the Hall device used to measure the MOC transport. Six Au contacts were used in parallel to measure the Hall voltage and the drain-source voltage. (b) Dependence on the scanning speed and the distance between the voltage peaks. The distance decreases as the measurement rate rises. The inset shows the drain-source *I*–*V* curve at room temperature, presenting hysteresis with opposite peaks; the distance between the peaks is denoted by dashed lines. ΔV is defined as the difference between the peaks’ distance at a certain rate and the fastest rate. (c) *I*–*V* curve with arrows indicating the direction of the voltage sweep. The device differs from the one presented in (a), although it shows similar behavior. (d) The Hall voltage shows that asymmetric characterization is dependent on the applied drain-source voltage and that spin plays a role in these devices. In all our D chiral devices, negative Hall sign is measured. (e) *I*–*V* characteristic curve of the device with a planar electrode configuration at different temperatures. (f) Temperature-dependent magnetic moment of the device. The plots present the magnetic moment measured when a potential of 0.5 V is applied and the magnetic moment measured at 0 V is subtracted. Inset: sketch of the device.

Figure 2e presents the temperature-dependent resistance, which indicates that, at a low temperature, when the crystal becomes antiferromagnetic (see ref (13)), the resistance increases, whereas at a higher temperature of above about 100 K the resistance follows the Arrhenius behavior, as shown in Figure 2f. From this plot, the activation energy can be obtained. This activation energy corresponds to the band gap of the material and was found to be 24 ± 2 meV, and independent of the current flow, as shown in Figures S1 and S2. At lower temperature, this material shows antiferromagnetic properties and it becomes ferromagnetic above 50 K. We relate the peak around *T* = 70 K to the phase transition (Figure 2e). It is important to know that the crystals are very stable under current and that no deterioration was observed when operating at 1 mA for many hours (Figure S3).

Three different types of chiral spintronic devices have been studied: a Hall device, a gated device, and a gated device with magnetic leads. The first configuration studied is the Hall configuration.^39^ As shown in Figure 3a, the Hall device contains six Au probes for measuring both the Hall and transport properties. The source-drain voltage is swiped from −15 to 15 V and back at different rates. The Hall voltage is measured in parallel with the drain-source current, enabling one to correlate spin accumulation with current. We ascribe the Hall signal to the anomalous Hall effect that results from scattering from the magnetic impurities.^39,40^ The symmetry is broken by the injection direction. The inset in Figure 3b shows that memristive behavior exists when the drain-source voltage is scanned. The nonlinear hysteresis loop of the spin MOC device differs from that of the standard oxide-based memristor. In the oxide-based memristor, the current applied increases the resistance, since the current fills the charge trap states. Here the device is based on magnetism induced by the current (similar to the light-induced magnetization explained above); therefore, the resistance is reduced by applying the current. Charging also affects the device, as can be seen by the nonzero current at zero voltage, after applying the voltage hysteresis loop. The combination of charging and the induced magnetism generates a strong dependence of the hysteresis on the current sweep frequency, and the hysteresis decays at a high frequency, similar to previously reported devices.^41^ This frequency dependence is presented in Figure 3b. With a higher sweep rate, the distance between the two hysteresis peaks is reduced.

Note that different devices exhibit similar behavior. For example, when comparing Figure 3b, c, and e, Figure 3c and d shows the drain-source and the Hall voltage hysteresis, respectively, for another device that was used to obtain the results shown in Figure 3b. The nonzero current at 0 V in Figure 3c, for example, is a result of charging due to the voltage applied on the crystals. This is in contrast to Figure 3e where a much smaller voltage was used. The Hall voltage response indicates that magnetization is generated when the current is driven. It is always negative, as expected when the spin polarization is due to the CISS effect, since the CISS effect generates the same magnetization when the current direction is switched.^29^ This occurs because the sign of the transported spin is reversed when the current direction is flipped.^21^ Note that nonuniform current by itself will generate a symmetric Hall response for the two current directions, which differs from the presented results (see also Figure S4 presenting the large difference between Rxx and Rxy).^42^ We ascribe the results to the CISS effect that creates an in-plane magnetization in the crystal. The crystal has a *P*21 space group with an in-plane chiral axis, and the current flows parallel to the surface of the Hall device. The moving electrons induce spin alignment of the unpaired electrons in the ions. This process results in strong in-plane magnetization with a small out-of-plane magnetization component near the contacts. A similar effect was shown, in our previous work,^13^ where the magnetic response is stronger when measured parallel to the crystal layers. The large correlation between the Hall signal hysteresis and the drain-source hysteresis could be exploited to reduce the device’s noise. It is important to mention that the memristor effect was observed in Cu-D crystals, as shown in Figure 3c, but not in Ni-L crystals, where we replaced the Cu atoms with Ni atoms (see Figures S7 and S8). The Ni ions have unpaired electrons as well; however, their ferromagnetic states are higher in energy, when compared to the Cu ions, and therefore thermal fluctuations and the current used are not enough to occupy them for the measurement time scale. These results demonstrate that the measured hysteresis in this system is related to the metal atoms and not to defects. In addition to the small magnetization induced in the MOC by thermal fluctuations,^13^ the spin current through the chiral potential polarizes the unpaired Cu electrons, inducing magnetization that is stabilized by exchange interactions. Our memrestive behavior differs from an oxide layer device, since here the resistance is reduced when current flows, due to the induced magnetization, while in oxide memristors the resistance increases with increasing current due to charging effect.

It was previously found that the phenylalanine crystals exhibit ferromagnetic behavior at room temperature and that the crystals become antiferromagnetic below 50 K.^13^ Therefore, if the hysteresis observed relates to the magnetic properties of the material, the hysteresis is expected to disappear at low temperatures. The temperature dependence of the *I*–*V* curves of a device is presented in Figure 3e. Indeed, the hysteresis gradually becomes smaller with decreasing temperature. This is also consistent with previous observations.^13^

Direct magnetization measurements, as a function of temperature, are presented in Figure 3f, along with a scheme of the device (inset). In these measurements, the residue signals from the substrate and the metal electrode were subtracted by deducting the signal obtained for source-drain voltages at 0.5 and 0 V. When the voltage was kept constant, the temperature was reduced from 300 to 3 K and then restored. With decreasing temperature, however, the difference in magnetization between the temperatures scanned up and down becomes smaller. Note that, upon reducing the temperature, the resistance of the device increases, especially at temperatures below 50 K; therefore, the current decreases. The observed temperature-dependent hysteresis is consistent with current-induced magnetization and is larger than spontaneous magnetization achieved by temperature fluctuations only. Below 50 K, the system becomes at least partially antiferromagnetic; therefore, magnetization is reduced.^13^ At temperatures above 50 K, the increase in magnetization is consistent with ferromagnetism. Upon warming, the magnetic moment is reduced, but the reduction is partially compensated by an increase in the current, resulting in increased magnetization. The temperature-dependent magnetic moment curve is controlled by two competing phenomena: (i) the magnetization induced by the current, which generates a metastable state, and (ii) the thermal magnetic state of the crystal. The combination of the two effects is responsible for the measured hysteresis loop.

The current-induced magnetization effect could be used to generate a multilevel spin memory, as displayed in Figure 4. The setup is shown in Figure 4a. The bottom gate is used to tune the density of the carriers in the system. The source is a magnetic Ni pad and the drain is a Au pad. A magnetic field of about 0.25 T was applied, using a permanent magnet in the direction of the current. The current levels are controlled by two in-plane magnetization directions: North and South and by random magnetization (0). In this way, it is possible to inject polarized spins into the crystal and to apply electric potential using the gate. Current versus voltage measurements were performed applying a magnetic field and with different gate voltages. To eliminate any residual magnetization of the Ni pads, before measurements, the sample was demagnetized by placing an out-of-plane magnetic field.

**Figure 4:**
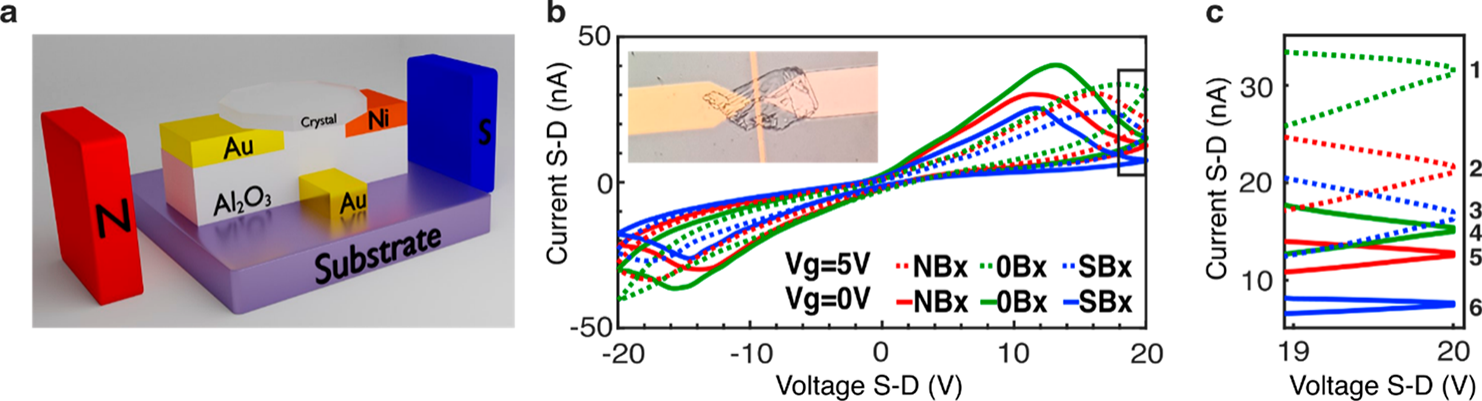
Multilevel 6 states for up, down, and no magnetization on the source pad. (a) Sketch of the bottom gate device and the measurement setup. Permanent magnets were placed along the current direction. (b) *I*–*V* dependency with two different gate voltages (0 V is denoted by solid lines and 5 V is denoted by dashed lines) and three different magnetic fields for each gate voltage (see also Figure S5). Inset: Microscope image of the bottom gate device with a crystal placed between the Ni and Au pads. (c) Close-up of a narrow voltage band of the *I*–*V* curves, as denoted in (b), showing the six different double states.

The multilevel logic is shown in Figure 4b and c, which presents examples of six double-level logic, under ambient conditions, at 0 and 5 V gate voltages. Each hysteresis loop of a specific external magnetic field and gate voltage represent a two levels system of high and low resistance. This system can be shifted both by magnetic field and by gate voltage to form a multilevel system. Nonuniform current induced by crystal defects decreases the efficiency of the effect. Applying an external magnetic field aligns all the spins and therefore adds a shift to the magnetoresistance curve. The parameters of the levels can be easily tuned by choosing a different drain-source voltage or gate voltage (see, for example, Figure S5 for 0 and 5 V gate voltage). Note that the direction of the external magnetic field applied does not coincide perfectly with the direction of the current-induced magnetization in the crystal. Consequently, the hysteresis observed is the largest if no external magnetic field is applied and the magnetic moment that exists is only due to the current-induced magnetization, with no interference of the external magnetic field.

The spin transistor behavior observed here, using the chiral d-phenylalanine-Cu crystals, results from the special properties of the crystal, which combine chirality and both ferromagnetism and ferroelectric properties at room temperature. The current through the chiral crystal is spin selective, due to the CISS effect. The spin current induces the magnetization of the unpaired electrons on the Cu^2+^ ions. Hence, the hysteresis in resistance and nonlinearity results from reducing scattering within the crystal, upon the formation of ferromagnetic domains. The copper ions are responsible for the relatively good conduction of the crystals at room temperature. As was observed by calculations, the ion states lie just above the highest lying molecular orbitals (HOMO) and indicate a barrier of about 20 meV for conduction. In addition, as shown in Figure 2, when the crystals are illuminated, the current is influenced by the circular polarization of the light. As a result of all these properties, the conduction through the crystals is susceptible to both electric and magnetic fields as well as to the spin direction of electrons injected from the magnetic source electrode, opening a wide range of control parameters for a two-terminal device (Figure 3). These control parameters enable one to achieve multilevel logic using simple organic materials (Figure 4).

The present work shows that, by using metal organic materials with the CISS effect, one can fabricate devices with new properties that are stable for a long time at ambient conditions. It is interesting to note that thermal induced magnetism was observed recently also in inorganic chiral crystals, and hence, the phenomena described here may appear in wide variation of chiral materials.^43^ The device presented shows dependence on the external magnetic field and a measurable Hall signal. The ability to combine chirality and magnetism with good conduction makes these crystals attractive candidates for new spintronic applications, when their size can be on the order of tens of nanometers.

## Supporting Information Available

Fabrication process, measurements methods, properties of the metal organic material, additional conduction and magnetic measurements, Arrhenius plot for activation energy at a higher temperature region estimation, transverse and longitudinal resistivity comparison, circular polarization calibration information (PDF)
