# Supplementary material for: Metal Organic Spin Transistor
Source: Nano Lett. 2021 Oct 18;21(20):8657–63. doi: 10.1021/acs.nanolett.1c01865 (PMC8859851; doi:10.1021/acs.nanolett.1c01865)
Supplement: Supplementary file 1 — nl1c01865_si_001.pdf [file nl1c01865_si_001.pdf]

**Supporting Information**

# Metal Organic Spin Transistor

*Naama Goren[1+], Tapan Kumar Das[2+], Noam Brown[3], Sharon Gilead[3], Shira Yochelis[1], Ehud Gazit[3]*, Ron Naaman[2]*, and Yossi Paltiel[1]**

1. Applied Physics Department and the Center for Nano-Science and Nano-Technology, The Hebrew University of Jerusalem, Jerusalem, 91904 Israel.
2. Department of Chemical and Biological Physics, Weizmann Institute, Rehovot, 76100 Israel.
3. Department of Molecular Microbiology and Biotechnology, The Shmunis School of Biomedicine and Cancer Research, George S. Wise Faculty of Life Sciences, Tel Aviv University, Tel Aviv 6997801, Israel.

**Corresponding Author**

Yossi Paltiel paltiel@mail.huji.ac.il

Ron Naaman Ron.Naaman@weizmann.ac.il

Ehud Gazit ehud.gazit@gmail.com

***D-phenylalanine-Cu crystal fabrication*:** The crystals were fabricated by reacting phenylalanine (10 mM), sodium hydroxide (20 mM), and $CuCl_2$ (10 mM) in water at 60°C. Blue plate-like crystals were formed during the cooling stage.

***High-resolution Scanning electron microscopy*:** The samples were drop cast on siliconized glass and dried at ambient conditions overnight. Finally, the samples were sputtered with chrome and imaged using a JSM-6700F High-Resolution Field Emission SEM (Jeol, Japan), operating at an acceleration voltage of 10 kV.

***Optical microscopy*:** The crystals were observed using a Nikon Eclipse Ti-E microscope.

***Superconducting Quantum Interference Device Measurements*:** Magnetization was measured using a commercial SQUID magnetometer (MPMS3, Quantum Design). The device for these measurements was fabricated on a thermal oxide ($SiO_2$-100 nm) p-type silicon wafer in a vertical architecture that was the same as was used with the MR device; however, only 200 nm of Au was used as the top electrode. The measurements were carried out with and without applying input voltage. The temperature-dependent magnetic moment was taken at a FCH mode ranging from 2K to 300K and vice versa. The difference in the magnetic moment between 0.5 V and 0 V was available for the analysis. The Keithley SMU model 2400 was used as the current source; the Keithley nanovoltmeter model 2182A was used to apply voltage and to measure current across the junction.

***Bottom gate transistor - device and measurements*:** A bottom gate transistor was fabricated. We evaporated a 10 μm-wide gate electrode with 10 nm Ti and 50 nm Au on a $SiO_2$ substrate. This was

followed by a 50 nm $Al_2O_3$ deposit by ALD as a gate barrier. On top, we evaporated the source (60 nm Ni and 10 nm Au) and drain (10 nm Ti and 60 nm Au) electrodes spaced 30 µm apart, so that the gate was located between them. Using wet etching, we accessed the bottom gate pad. Finally, the crystals were placed manually, and the device was connected by a wire bond. Measurements were carried out at room temperature. We used crystals with surface size of about 100X200 µm and with a thickness of about 300 nm, with voltages between 0 to 20V. The current density was about 100A/m^2.

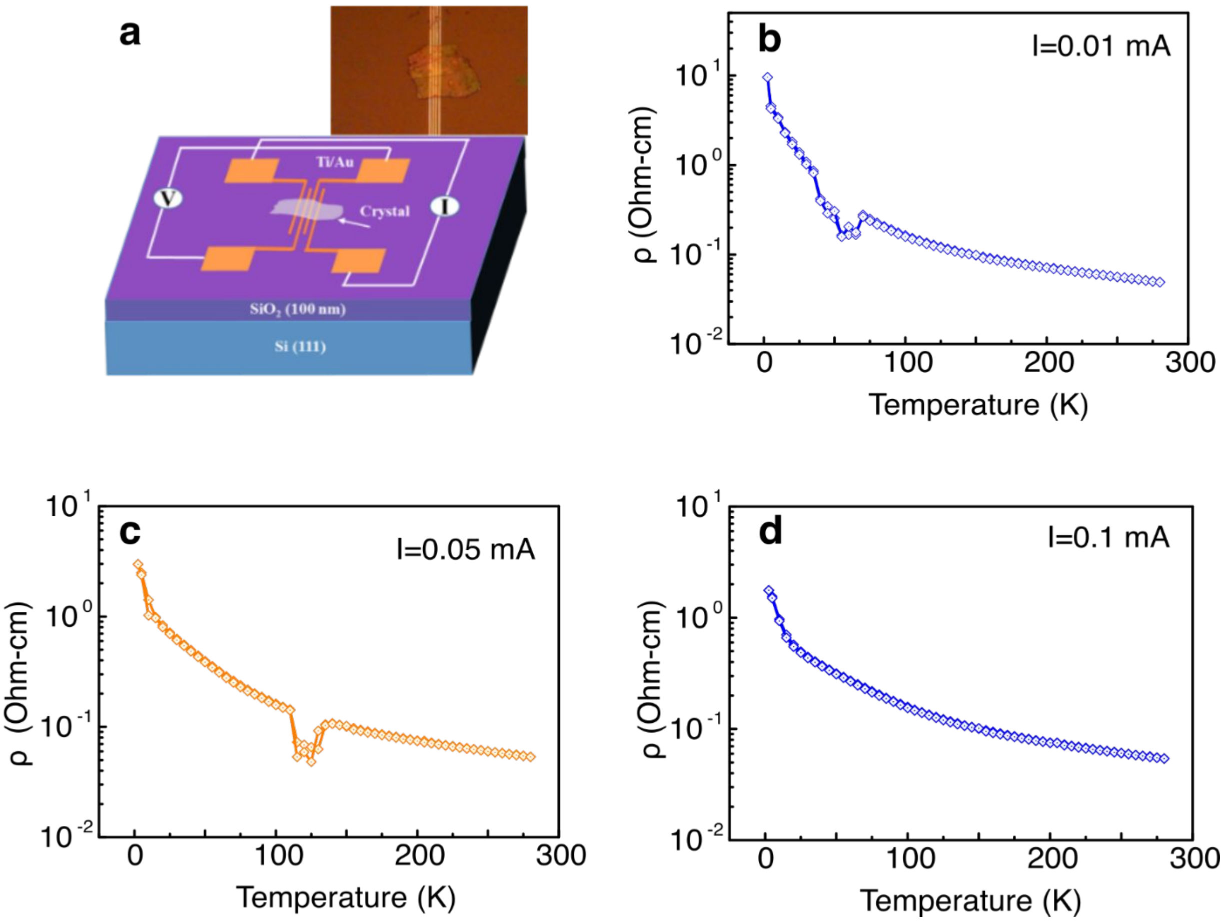


**Figure S1.** Conduction measurement. (a) Schematic of the planner device with four electrodes; the crystal was placed on top of the Au-pad (see an optical image of a crystal on the pad, inset). The surface resistance measured by four points contacts with a different input current; (b) 0.01 mA, (c) 0.05 mA, and (d) 0.1 mA, respectively present in log scale. These materials, show antiferromagnetic properties at low temperatures and becomes ferromagnetic above 50K. We relate the feature around T=70K to the phase transition.

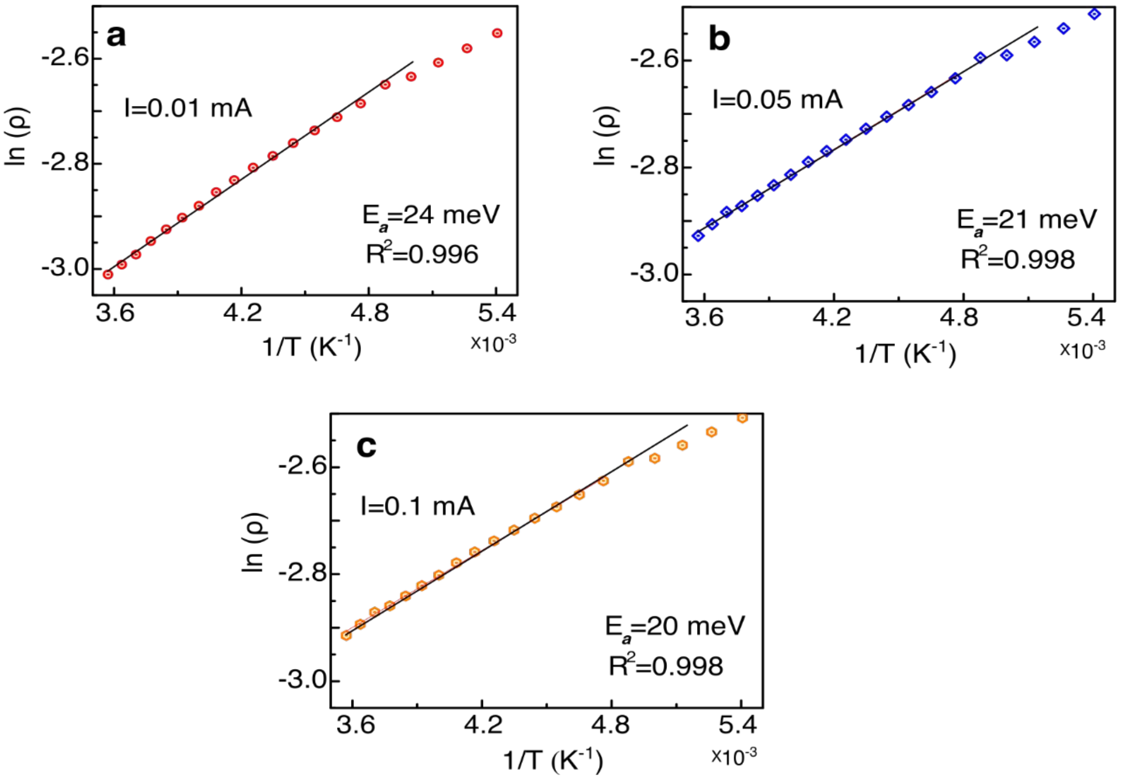


**Figure S2.** Activation energy. Arrhenius plot for activation energy at a higher temperature region estimated from the conduction plots with different input current. (a) 0.01 mA, (b) 0.05 mA, and (c) 0.1 mA, respectively.

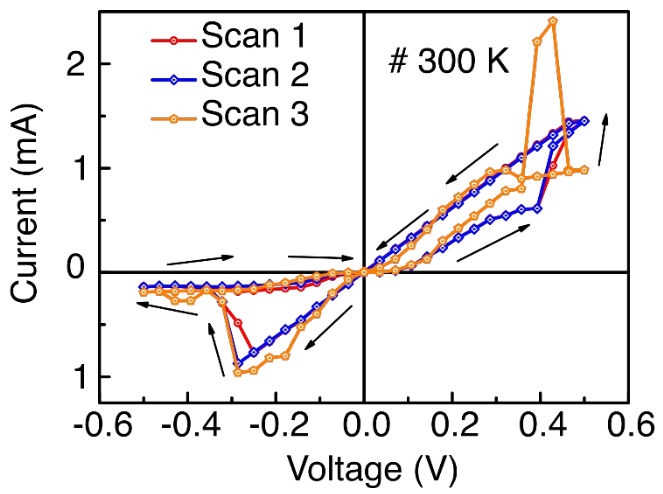


**Figure S3.** Memristor device characteristics. The current-voltage characteristic curve of the device with a planar electrode configuration at room temperature with a multiple scan.

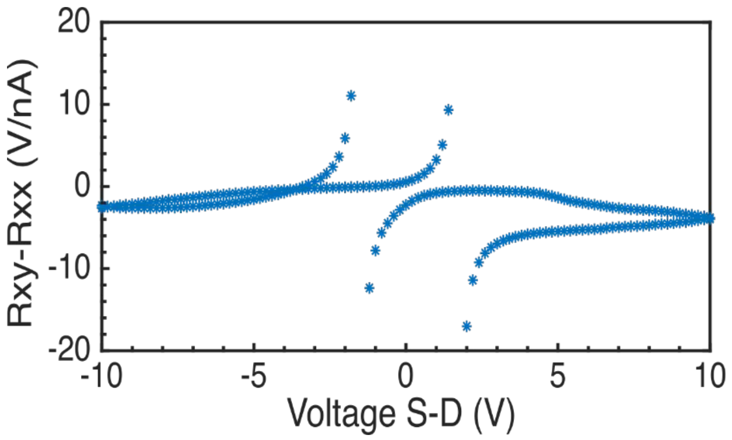


**Figure S4.** The difference between Rxx and Rxy as a function of drain source voltage. The non-symmetric curve suggests that there are no simple correlations between the two resistance values.

.

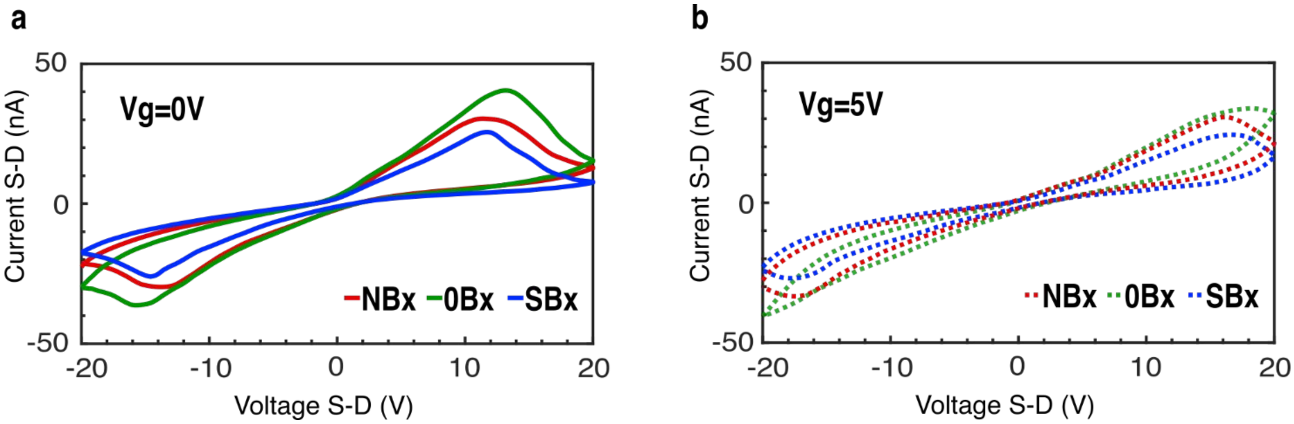


**Figure S5.** Multilevel system. Current-volt measurements of a double level system with three different magnetic fields (up, down, and none), which become a six-level system for each gate voltage: (a) zero gate voltage and (b) 5V gate voltage.

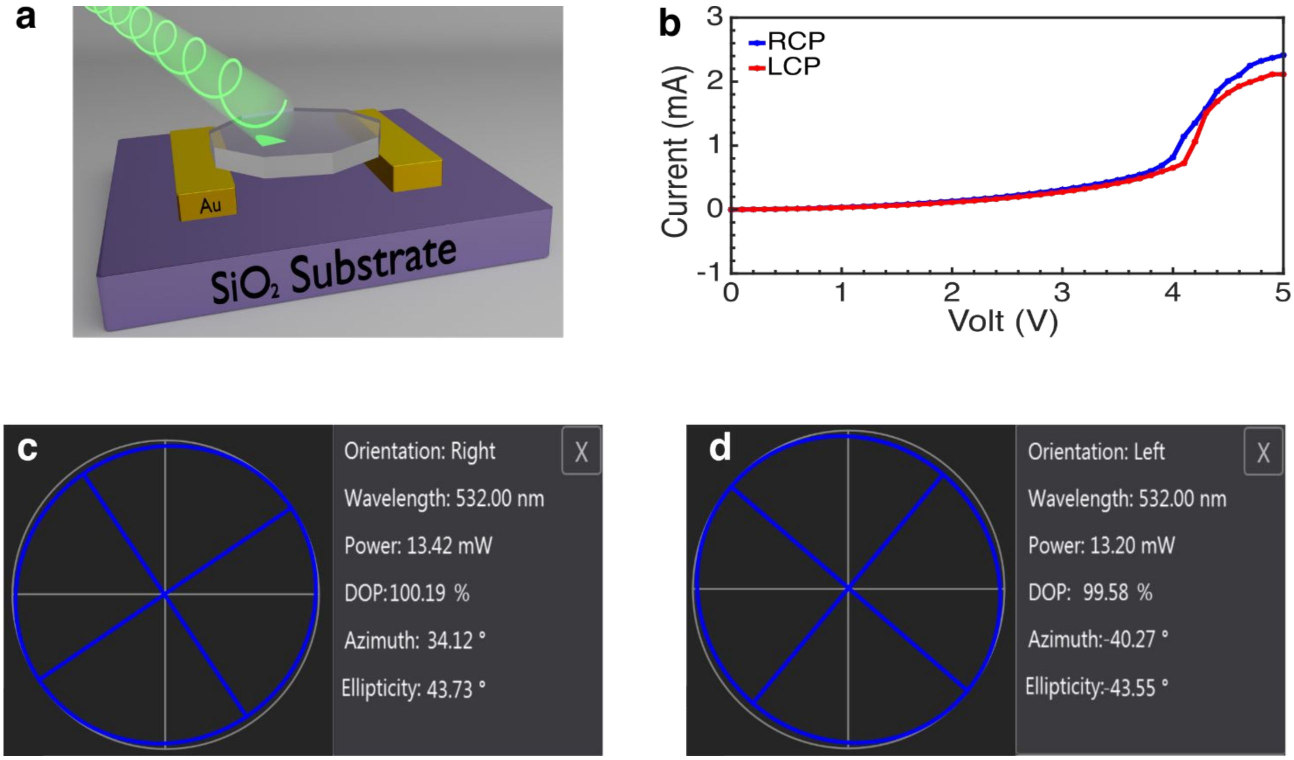


**Figure S6.** Photocurrent device. (a) Sketch of the device and the set-up of the photocurrent measurement. A crystal placed above two gold (Au) contacts; the current was measured while a constant 5V voltage was applied and circular polarized light (532 nm) was illuminated. (b) IV curve of the device under illumination. Calibration of right (c) and left (d) circular polarization degree. It is interesting to note that the response for both polarizations change with bias voltage and is not linear. For example, at 4.5V the currents for both polarizations become similar. This is a key feature of the CISS. The CISS is not a single electron process, rather it is strongly depending on multiple electron polarization. Therefore, both the memory effects, the bias voltage, and the resulting polarization are expected to change the CISS efficiency, as seen for the different bias voltages.

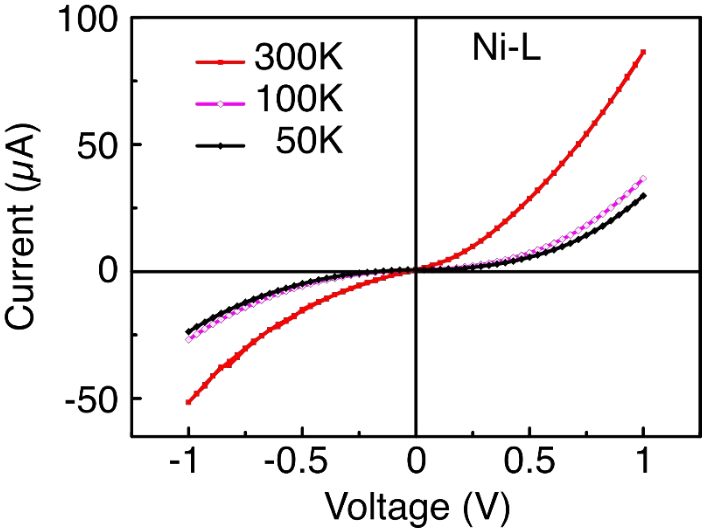


**Figure S7.** IV curve of the Ni-L crystal in different temperatures. Hysteresis was not observed for this type of crystal.

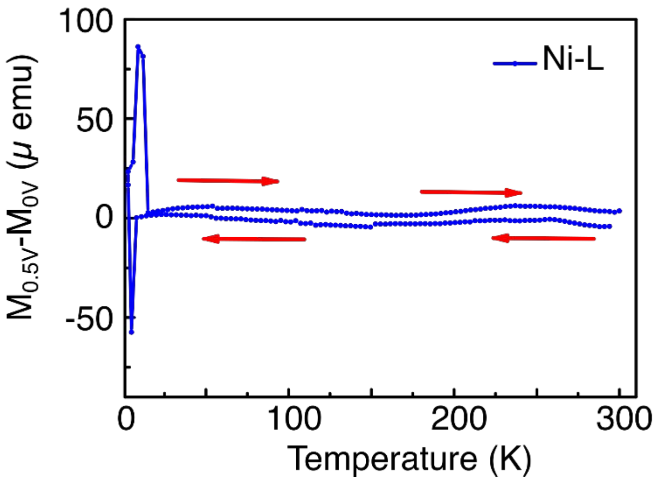


**Figure S8.** Superconducting Quantum Interference Device Measurements of the Ni-L crystal.
